# Serum S100B in primary progressive multiple sclerosis patients treated with interferon-beta-1a

**DOI:** 10.1186/1477-5751-3-4

**Published:** 2004-10-13

**Authors:** Ee Tuan Lim, Axel Petzold, Siobhan M Leary, Daniel R Altmann, Geoff Keir, Ed J Thompson, David H Miller, Alan J Thompson, Gavin Giovannoni

**Affiliations:** 1 Department of Neuroinflammation, Institute of Neurology, University College London, Queen Square, London, WC1N 3BG, UK; 2 NMR Research Unit, Institute of Neurology, University College London, Queen Square, London, WC1N 3BG, UK; 3 Medical Statistics Unit, London School of Hygiene & Tropical Medicine, Keppel Street, London, WC1E 7HT, UK

**Keywords:** serum S100B, primary progressive multiple sclerosis, Interferon β-1a, magnetic resonance imaging

## Abstract

S100B belongs to a family of calcium-binding proteins implicated in intracellular and extracellular regulatory activities. This study of serum S100B in primary progressive multiple sclerosis (PPMS) is based on data obtained from a randomized, controlled trial of Interferon β-1a in subjects with PPMS. The key questions were whether S100B levels were associated with either disability or MRI findings in primary progressive MS and whether Interferon β-1a has an effect on their S100B levels. Serial serum S100B levels were measured using an ELISA method. The results demonstrated that serum S100B is not related to either disease progression or MRI findings in subjects with primary progressive MS given Interferon β-1a. Furthermore there is no correlation between S100B levels and the primary and secondary outcome measures.

## Introduction

S100B belongs to a family of calcium-binding proteins implicated in intracellular and extracellular regulatory activities [2]. Intracellularly, it exhibits regulatory effects on cell growth, differentiation, cell shape and energy metabolism. Extracellularly, S100B stimulates neuronal survival, differentiation, astrocytic proliferation, neuronal death via apoptosis, and stimulates (in some cases) or inhibits (in others) activity of inflammatory cells.

Several studies suggest that S100B has a role in the pathogenesis of multiple sclerosis (MS). Phenotypically and functionally similar T cells specific against S100B can be detected in the peripheral blood of MS patients making S100B a putative candidate auto-antigen in MS [15]. Furthermore, S100B may act as a cytokine [2,10,11] and in vitro studies show that, at high levels, S100 can induce the neuronal expression and secretion of pro-inflammatory interleukin-6. In addition, elevated levels of S100B have been detected in the cerebrospinal fluid (CSF) of MS patients during acute phases or exacerbations of the disease [10] and it has therefore been proposed that elevated S100B may be indicative of active cell injury [11].

Interferon-β (IFN-β) is effective in reducing relapse rate in relapsing-remitting [6,14,17] and secondary progressive MS [3] but the mechanisms behind the beneficial action of IFNβ are not fully understood. Two potential sites of action are on cytokine production [1,4,12] and on the entry of leukocytes into the CNS [8,9,16,18].

In this clinically negative phase II study [7], we assessed the effect of IFNβ-1a on serum levels of S100B at 3-month intervals in subjects with primary progressive MS (PPMS). The key questions were whether serum S100B levels correlated with disability or MRI findings in patients with PPMS, and whether IFN-β has an effect on levels of serum S100B.

## Methods

### Patients and examination

**Figure 1:**
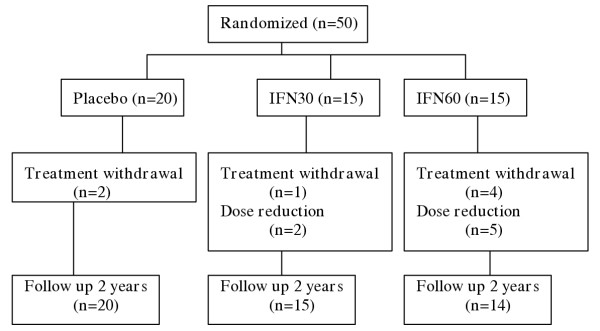
Fifty subjects with PPMS were randomised in a phase II trial of Interferon β-1a and were assessed 3 monthly over a 2-year study period. n = number of subjects with PPMS

Fifty patients with PPMS were recruited in a phase II trial of IFNβ-1a (Avonex^®^, Biogen) and were assessed three monthly over a study period of 2 years. Fifteen of these patients were treated with IFNβ-1a 30μg intramuscularly (im) weekly (IFN30), 15 received IFNβ-1a 60μg im weekly (IFN60) and 20 with placebo. IFNβ-1a was reduced to half dose in 5 subjects receiving 60μg im weekly, and in 2 subjects receiving IFNβ-1a 30μg im weekly. Seven subjects withdrew from treatment [7] (see Figure 1).

Neurological examination was performed at each visit and disability was measured using Kurtzke's expanded disability status scale (EDSS). Progression was defined as a sustained (3 months apart) increase of at least 1.0 on the EDSS scale between 0 to 5 and 0.5 for subjects with EDSS score of 5.5 and above.

Fourteen healthy subjects served as controls.

All subjects provided informed consent prior to their inclusion in the study. This study was approved by the ethics committee and has therefore been performed with the ethical standards laid down in the 1964 Declaration of Helsinki.

### MR imaging and analyses

MRI was performed at baseline and 6 monthly for 2 years. Only baseline and year 2 data were included in this study. Brain and spinal cord atrophy, ventricular volume, T1 and T2 lesion load were measured as described elsewhere [7].

### Serum S100B levels

Serum samples were centrifuged and stored at -20°C. Serum S100B levels were quantified using a modified ELISA method as previously described by Green et al. [5]. Ninety-six-well plates were coated with 100μl 0.05 M carbonate buffer containing 10μl monoclonal anti-S100B (Affiniti Research Products, Exeter, UK). The plates were washed with 0.67 M barbitone buffer containing 5 mM calcium lactate, 0.1% BSA and 0.05% Tween and then blocked with 2% BSA and washed again. Diluted serum (1:1) in 0.67 M barbitone buffer containing 5 mM calcium lactate was added in duplicate. After incubation and wash 0.1% HRP conjugated polyclonal anti-S100B (Dako, Copenhagen, Denmark) was used as detecting antibody. The OPD colour reaction was stopped with 1 M hydrochloric acid and the absorbance read at 492 and 405 nm. The antigen concentration was calculated against a standard curve ranging from 0.01 to 2.5 ng/ml.

### Statistical analyses

Median, interquartile range and significance of group differences (Mann-Whitney U tests) were evaluated. Changes of serum level over time were examined using variance components regression models of serum response variable on time as predictor, with random subject-specific intercepts and fixed common slopes. Curvature was assessed using a quadratic term in time; modification of curve over time by treatment was assessed using additional terms for treatment and treatment by time interaction in the model. Two sets of treatment terms were used: i) indicators of assigned weekly dose ii) average weekly dose over follow-up (including changes to dose regime) as continuous variable. Modification of the curve over time by MRI variable values were similarly examined using terms for MRI variable and MRI variable by time interaction.

Direct associations between serum level and MRI/clinical variables were examined by regression models of 24 month serum on 24 month MRI variable, adjusting for baseline serum and MRI values (this type of model takes into account change from baseline), with additional terms for treatment and treatment by MRI variable interaction, the latter to assess possible modifications of the relationship by treatment.

Software used were the SPSS software package (version 11.0 for Windows) and Stata 7.0 (Stata Corporation. Stata Statistical Software: Release 7.0. College Station, Texas, USA).

## Results

### Serum S100B between subjects with PPMS and controls

The median and interquartile ranges for all subjects are described in Table 1. There were no significant differences between any of the groups in relation to age. When comparing S100B levels at baseline of subjects with PPMS and controls, the difference was not statistically significant (p = 0.3).

**Table 1:** Age and serial serum S100B levels expressed as median (interquartile range). n = number of subjects; mo, months; N/A, non-applicable.

	**Control (n = 14)**	**Placebo (n = 20)**	**IFN30 (n = 15)**	**IFN60 (n = 15)**
Male:Female	6:8 n = 14	15:5 n = 20	10:5 n = 15	7:8 n = 15
Age (years)	32 (29–44) n = 14	43 (36–51) n = 20	51 (39–53) n = 15	52 (43–54) n = 15
S100B-0mo	0.08 (0.08–0.10) n = 14	0.09 (0.02–0.10) n = 20	0.06 (0.04–0.10) n = 12	0.07 (0.04–0.10) n = 14
S100B-3mo	N/A	0.08 (0.05–0.10) n = 20	0.06 (0.05–0.10) n = 15	0.07 (0.04–0.10) n = 14
S100B-6mo	N/A	0.10 (0.03–0.20) n = 18	0.07 (0.04–0.10) n = 15	0.07 (0.04–0.10) n = 14
S100B-9mo	N/A	0.08 (0.03–0.10) n = 18	0.05 (0.02–0.10) n = 15	0.06 (0.04–0.10) n = 14
S100B-12mo	N/A	0.07 (0.03–0.10) n = 17	0.08 (0.05–0.10) n = 15	0.08 (0.04–0.10) n = 14
S100B-15mo	N/A	0.07 (0.02–0.10) n = 19	0.07 (0.04–0.10) n = 14	0.09 (0.04–0.10) n = 14
S100B-18mo	N/A	0.07 (0.04–0.10) n = 18	0.06 (0.02–0.09) n = 13	0.09 (0.03–0.20) n = 14
S100B-21mo	N/A	0.06 (0.05–0.10) n = 18	0.08 (0.04–0.10) n = 14	0.08 (0.04–0.20) n = 13
S100B-24mo	N/A	0.07 (0.02–0.10) n = 18	0.07 (0.05–0.10) n = 15	0.06 (0.04–0.10) n = 13

### Serum S100B change over time

There was no change over time in the serum S100B levels. The shape of the serum trajectory did not vary between the treatment regimes, i.e. placebo vs. IFN30 vs. IFN60.

### Serum S100B versus Clinical and MRI parameters (Table 2)

There was no evidence that the 24-month serum S100B values were associated with either changes in the T1 or T2 loads, or ventricular or cord volumes at 24 months, after adjusting for the baseline values of each subject. There was no correlation with disease progression on the EDSS. There was also no evidence that these relationships were modified by treatment assignment (intention-to-treat analysis) (Table 2) or the overall average dose, which included the changes to treatment regime (non-intention-to-treat analysis) (Table 2).

**Table 2:** Serum S100B versus Clinical and MRI variables. Estimated mean change in 24-month serum S100B associated with unit increase in mean value of T1 and T2 lesion load, ventricular and spinal cord volume, adjusted for baseline values of both serum S100B and of MRI parameters. Baseline adjustment ensures that the coefficient assesses the 'effect' of the 24-month MRI parameters value relative to its baseline. * Test of treatment interactions with row variable.

**Variable**	**Coefficient**	**P-value**	**95% Confidence Interval (CI)**	P-value for treatment modification*: Assignment average dose	
24 month T1 load	-4 × 10^-6^	0.35	-1 × 10^-5^, 4 × 10^-6^	0.76	0.59
24 month T2 load	-3 × 10^-6^	0.16	-7 × 10^-6^, 1 × 10^-6^	0.57	0.89
24 month ventricular volume	7 × 10^-7^	0.75	-3 × 10^-6^, 5 × 10^-6^	0.46	0.24
24 month cord volume	-2 × 10^-3^	0.54	-9 × 10^-3^, 5 × 10^-3^	0.58	0.88

## Discussion

These results suggest that serum S100B levels in patients with PPMS were not affected by intramuscular IFNβ-1a and that there was no observable change in S100B over time. Furthermore, we did not observe any correlation between S100B levels and clinical disability or between S100B and quantitative MRI measures.

This study therefore suggests Although there is evidence that S100B elevation in MS is related to inflammatory activity [10,11,13], this study has shown that S100B was not sensitive to disease progression in PPMS. This supports the view that PPMS is less inflammatory than other forms of MS and that serum S100B would be ineffective as a surrogate marker of disease progression in this subgroup.

It would be valuable to identify surrogate markers of clinical progression in PPMS to aid the development of effective therapeutic intervention, since clinical trials with a disability endpoint are very large and resource consuming. It is possible that such markers would need to be less related to acute inflammation and more dependant on other neuropathology such as axonal loss and regeneneration.
